# *Portulaca oleracea* polysaccharides reduce serum lipid levels in aging rats by modulating intestinal microbiota and metabolites

**DOI:** 10.3389/fnut.2022.965653

**Published:** 2022-08-02

**Authors:** Qiang Fu, Hui Huang, Aiwen Ding, Ziqi Yu, Yuping Huang, Guiping Fu, Yushan Huang, Xiaoliu Huang

**Affiliations:** ^1^College of Medicine, Jinggangshan University, Ji’an, China; ^2^Institute of Spinal Diseases, Jinggangshan University, Ji’an, China; ^3^Department of Biochemistry and Molecular Biology, Gannan Medical University, Ganzhou, China; ^4^Center for Evidence Based Medical and Clinical Research, First Affiliated Hospital of Gannan Medical University, Ganzhou, China

**Keywords:** *Portulaca oleracea* polysaccharides, serum lipid level, aging rats, intestinal microbiota, metabolites

## Abstract

Metabolic diseases characterized by dyslipidemia are common health problems for elderly populations. Dietary fiber intake is inversely associated with the risk of dyslipidemia. This study investigated the effects of *Portulaca oleracea* polysaccharide (POP) on the intestinal microbiota and its metabolites in aging rats using 16S rRNA sequencing and metabolomics techniques. Our results showed that POPs reduced the ratio of Firmicutes/Bacteroidetes (F/B), relative abundance of Fusobacteria, and levels of triglyceride (TG), low-density lipoprotein cholesterol (LDL-C), alanine aminotransferase (ALT), and gamma-glutamyl transferase (γ-GT) in the serum of aging rats. POP supplementation also reduced 5beta-cholestane-3alpha,7alpha,12alpha,25-tetrol, and vaccenic acid concentrations in lipids and lipoid-like molecules, while soyasapogenol E and monoacylglycerol (MG) (24:0/0:0/0:0) levels increased. This study demonstrated that POP’s beneficial effects on lipid levels in aging rats might be partially attributable to the modification of gut microbiota and related metabolites.

## Introduction

Age is one of the main physiological factors that increases serum lipid levels, which leads to increased levels of triglyceride (TG), serum total cholesterol (TC), low-density lipoprotein cholesterol (LDL-C), and decrease of high-density lipoprotein cholesterol (HDL-C); all of these are dyslipidemia characteristics (1, 2). TC and LDL-C levels increased with normal aging, while HDL-C decreased with it. Elevated plasma LDL-C levels are one of the most important risk factors for atherosclerotic cardiovascular disease (ASCVD) (1, 3, 4). By 2050, China will have approximately 400 million citizens aged >65 years, including 150 million aged >80 years. It is apparent that China faces a huge healthcare challenge owing to its aging population (5, 6).

Dysregulation of the intestinal microbiota and its metabolites relates to the development of metabolic diseases, such as hyperlipidemia, diabetes, hypertension, and obesity (7, 8). A study on elderly patients with coronary heart disease showed an association between increased blood lipid levels and abnormal intestinal microbiota distribution, which may influence patients’ serum lipid levels (9). Animal experiments have also revealed a significant decline in the relative abundance of *Bilophila*, bile salt hydrolase-rich *Bifidobacterium*, and *Lactobacillus*. Meanwhile, decreases in deoxycholic acid and lithocholic acid are potential lipid-lowering mechanisms for improving lipid disorders (10).

Diet is one of the most important factors affecting gut microbiome composition, particularly indigestible food components (11, 12). Dietary fiber has anti-digestion and anti-absorption properties and is mainly fermented by the intestinal microbiota, which affects its composition and function (13, 14). In one study, significant differences in microbial function among adults in the United States, Malawi, and Venezuela were attributed to protein (carnivorous) and dietary fiber consumption (herbivorous), respectively (12). In Western societies, low fiber intake is a driving factor in the reduction of human gastrointestinal microbiota, increasing the risk of chronic non-communicable diseases, such as obesity, cardiovascular disease, and type 2 diabetes (14, 15). Thus, the dietary reference intake recommends a daily allowance of 38 g/day of dietary fiber in men and 25 g/day in women aged 19–50 years. Men and women aged >51 years should consume 31 g/day and 21 g/day of dietary fiber, respectively (16). The regulation of the production of gut microbiota and bacteria-derived metabolites is attributed to the beneficial effects of dietary fiber on dyslipidemia (17, 18). Bioactive polysaccharides also improve dyslipidemia and reduce the risk of cardiovascular disease (16). Previous studies have found that polysaccharides from *Portulaca oleracea* (POPs) help regulate lipid metabolism in diabetic mice (19). However, its effect on lipid levels in elderly individuals is unclear. This study aimed to investigate the structural changes in the intestinal microbiota, map the bacteria-derived metabolites, and understand the effect of POPs on lipid levels in elderly populations.

## Materials and methods

### Equipment and reagents

The equipment and reagents used in this study included: High Resolution Mass Spectrometer (QE Plus, Thermo Fisher Technologies, Waltham, MA, United States), PCR Amplifier (580BR10905, Bio-Rad, Hercules, CA, United States), Bioanalyzer (2100, Aglient, Santa Clara, CA, United States), NanoDrop (2000, Thermo Fisher, Waltham, MA, United States). High performance liquid chromatography (Dionex U3000 UHPLC, Thermo Fisher Technologies, Waltham, MA, United States). A chromatographic column, (100 mm × 2.1 mm, 1.8 μm), was purchased from Waters (ACQUITY UPLC HSS T3, Milford, MA, United States). A DNeasy PowerSoil Kit (Cat. No. 12888) and QIAamp 96 PowerFecal QIAcube HT kit (Cat. No. 51531) was purchased from QIAGEN (Germantown, MD, United States). Qubit dsDNA Assay Kit (Cat. No. Q32854) was purchased from Life Technologies (Eugene, OR, United States). A Tks Gflex DNA Polymerase (Cat. No. R0608) was purchased from Takara (Dalian, China). All chemicals and solvents were analytical or HPLC-grade. Water, methanol, acetonitrile, and formic acid were purchased from CNW Technologies GmbH (Düsseldorf, Germany). L-2-chlorophenylalanine was purchased from Shanghai Hengchuang Biotechnology Co., Ltd. (Shanghai, China).

### Proportion of feed purslane

Purslane polysaccharides (≥50.0%) and purslane powder were purchased from Lanzhou Wotelaisi Biotechnology Co., Ltd. (Wotls, Lanzhou, China). The pellet feed for the experiments’ rats was purchased from Hunan SJA Laboratory Animal Co. Ltd [Production License No. SCXK(Xiang)2014-0002]. A POP supplemented diet was prepared by combining 1.0% POPs with 99.0% pellet feed. The whole *P. oleracea* herb supplemented diet was prepared by adding 3.5% *P. oleracea* powder to 96.5% pellet feed.

### Animals and experiment design

Thirty 18-month-old SPF-grade SD rats were purchased from Hunan SJA Laboratory Animal Co. Ltd. [Laboratory animal production license, SCXK(Xiang)2019-0004]. All animals were acclimatized at 23 ± 1°C, 50–60% relative humidity, and 12/12 h light/dark cycle for 1 week before the animal experiments commence. The rats (*n* = 10 per group) were divided into the Con (normal rat pellet feed), POP (1.0% POPs added to 99.0% pellet feed), and herb (3.5% *P. oleracea* powder combined with 96.5% pellet feed) groups. The rats ate and drank freely. Food intake and fasting blood glucose were recorded on the last day of each week. At the end of the 15-week experimental period, the rats were sacrificed by heart puncture after anesthesia with urethane, and biological samples were collected for further analysis. The Animal Care and Use Committee of Jinggangshan University [SYXK (Gan) 2017-0003] approved all animal use procedures.

### Metabolomic analysis of the gut microbiota

A day before the end of the experiment, the animals were held in aseptic conditions, and their anuses were squeezed to stimulate defecation. The fecal samples were collected with aseptic cryopreservation tubes and stored at −80°C after liquid nitrogen quick-freezing.

Approximately 60 mg of intestinal content was added to 20 μL of L-2-chlorophenylalanine (0.3 mg/mL, methanol configuration) and 600 μL methanol-water (v:v = 4:1). Then, they were ground for 2 min, ultrasonically extracted for 10 min in an ice-water bath, and centrifuged for 10 min at 13,000 rpm at 4°C. A volume of 300 μL of supernatant air dried and then added to 400 μL of methanol-aqueous solution for redissolving, followed by vortex and ultrasonication for 3 min. It was then centrifuged at 13,000 rpm for 10 min at 4°C, and filtered (0.22 μm). UPLC-Orbitrap/MS analysis was performed later.

The stool samples were analyzed using a UPLC-Orbitrap/MS (Thermo Scientific™ Q Exactive™ Plus). Liquid chromatographic separation occurred on a Dionex Ultimate 3000 RS UHPLC system (Thermo Fisher Scientific, Waltham, MA, United States). Chromatographic separation was performed with an ACQUITY UPLC HSS T3 column (100 mm × 2.1 mm, 1.8 μm) at 45°C. The flow rate was set at 0.35 mL/min and the injection volume was set at 5.0 μL. The mobile phase consisted of distilled water (A, containing 0.1% formic acid, v/v) and acetonitrile (B, containing 0.1% formic acid, v/v) for the positive and negative modes. The binary gradient program was applied as follows: 0–2 min, 5% B; 2–4 min, 5–25% B; 4–8 min, 25–50% B; 8–10 min, 50–80% B, 10–14 min, 80–100% B, 14–15 min, 100% B, 15–15.1 min, 100–5% B; 15.1–16 min, 5% B. The mass ranged from 100 to 1,000 m/z. The resolution was set at 70,000 for the full Mass Spectrometry (MS) scans and 17,500 for the HCD MS/MS scans. The collision energy was set at 10, 20, and 40 eV. The mass spectrometer operated as follows: spray voltage, 3,800 V (+) and 3,000 V (−); sheath gas flow rate, 35 arbitrary units; auxiliary gas flow rate, 8 arbitrary units; capillary temperature, 320°C. The quality controls (QCs) were injected at regular intervals (every 10 samples) throughout the analytical run to provide a set of data from which repeatability could be assessed.

The acquired LC-MS raw data were analyzed using Progenesis QI software (Waters Corporation, Milford, MA, United States). Metabolites were identified using Progenesis QI (Waters Corporation, Milford, MA, United States) data processing software, based on public databases, such as http://www.hmdb.ca/, http://www.lipidmaps.org/, and self-built databases. The positive and negative data were combined to and imported into the R ropls package. Principle component analysis (PCA) and (orthogonal) partial least-squares-discriminant analysis (O) PLS-DA were carried out to visualize the metabolic alterations among the experimental groups, after mean centering (Ctr) and Pareto variance (Par) scaling, respectively. Hotelling’s T2 region, shown as an ellipse in the models’ score plots, defines the 95% confidence interval (CI) of the modeled variation. Variable importance in the projection (VIP) ranks the overall contribution of each variable to the orthogonal partial least squares discriminant analysis (OPLS-DA) model, and those variables with VIP > 1 are relevant for group discrimination.

In this study, the default 7-round cross validation was applied, with 1/7 of the samples being excluded from the mathematical model in each round to guard against overfitting.

The differential metabolites were selected on the basis of a combination of statistically significant thresholds of variable influence on projection (VIP) values obtained from the OPLS-DA model and *P*-values from a two-tailed Student’s *t*-test on the normalized peak areas, where metabolites with VIP > 1.0 and *P* < 0.05 were considered differential metabolites.

### Gut microbiota analysis

Total genomic DNA was extracted using a DNeasy PowerSoil Kit (QIAGEN, Germantown, MD, United States) following the manufacturer’s instructions. The DNA concentration was verified using NanoDrop and agarose gel. The V3–V4 variable regions of the 16S rRNA genes were amplified using universal primers 343F (5′-TACGGRAGGCAGCAG-3′) and 798R (5′-AGGGTATCTAATCCT-3′) and then sequenced using the Illumina Miseq platform from Shanghai OE Biotech Co., Ltd. (Shanghai, China). Raw sequencing data were in FASTQ format. Paired-end reads were preprocessed using Trimmomatic software to detect and remove ambiguous bases (N). It also removed low-quality sequences those with average quality scores below 20 using the sliding window trimming approach. After trimming, the paired-end reads were assembled using FLASH software. The assembly parameters were as follows: 10 bp of minimal overlapping, 200 bp of maximum overlapping, and 20% of maximum mismatch rate. Further denoising of the sequences was performed as follows: reads with ambiguous homologous sequences and those below 200 bp were abandoned. Reads with 75% of the bases above Q20 were retained. Then, chimeric reads were detected and removed. These two steps were achieved using QIIME software (version 1.8.0). Clean reads were subjected to primer sequence removal and clustering to generate operational taxonomic units (OTUs) using VSEARCH software with a 97% similarity cutoff. Each OUT’s representative read was selected using the QIIME package. All representative reads were annotated and blasted against the Silva database version 123 (or Greengenes) (16s/18s rDNA) using an RDP classifier (the confidence threshold was 70%). All representative reads were annotated and blasted against the UNITE database (ITSs rDNA) using BLAST.

### Statistical analysis

The statistical analysis was performed using SPSS 19.0 (SPSS Inc., Chicago, IL, United States). One-way analysis of variance (ANOVA) for multiple comparisons, followed by the LSD *t*-test, was used to determine the significance of differences between groups. A difference of *P* < 0.05 was significant. The data were expressed as ($\bar{x}$ ± s), and the profiles were plotted in GraphPad Prism, version 8.0 (GraphPad Software, Inc., La Jolla, CA, United States).

## Results

### Relative feed intake and blood glucose changes

**FIGURE 1:**
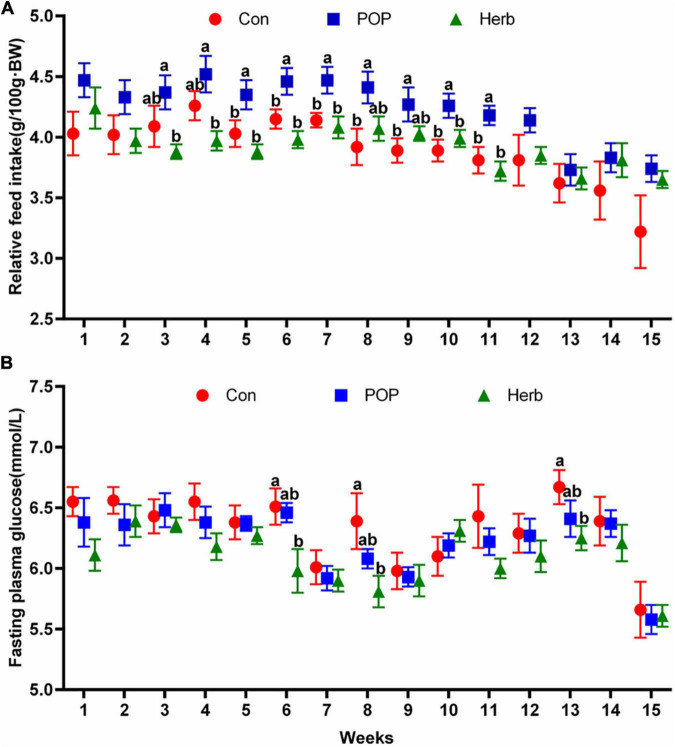
Relative feed intake **(A)** and fasting plasma glucose level **(B)** measurements after supplementation with POP and purslane. The different letters indicate significant differences (*P* < 0.05).

As age and digestive function decline, food intake amounts in aged individuals are affected. Additionally, abnormal glucose metabolism-related diseases are common problems in these people. Our results in aging rats showed that a POP-supplemented diet could improve the dietary intake in the aging rats, and the relative feed intake from week 5 to week 11 significantly increased compared with the control (Con) group; However, the purslane-supplemented diet (Herb) had no similar effect (Figure 1A). The results showed that POPs and the purslane could reduce blood glucose in aging rats, and the effect of the purslane was better than that of POP. Blood glucose levels in the herb group were significantly reduced at weeks 6, 8, and 13 compared with that of the Con group (Figure 1B).

### Body weight, organ index, blood lipid levels, and liver function index changes

**FIGURE 2:**
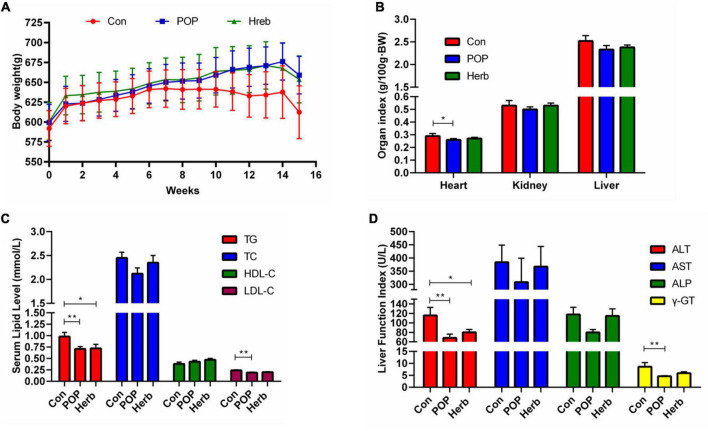
Effects of POP administration on **(A)** body weight, **(B)** organ index, **(C)** enzyme activity of liver function, and **(D)** serum lipid levels. The symbols * and ** indicate statistically significant differences among the groups (*P* < 0.05 and *P* < 0.01, respectively).

The body weight, organ index, blood lipid level (TG, TC, HDL-C, and LDL-C), and liver function results are shown in Figure 2. Compared with the Con group, POPs and *P. oleracea* showed a trend in preventing weight loss in aging rats, but no statistical difference was observed between the groups (Figure 2A). The purslane polysaccharide diet significantly reduced the cardiac organ index levels (TG, LDL-C, ALT, and γ-GT) in aging rats, while the purslane herb diet only significantly reduced TG and ALT levels (Figures 2B–D).

### Pathological examination of the colon

**FIGURE 3:**
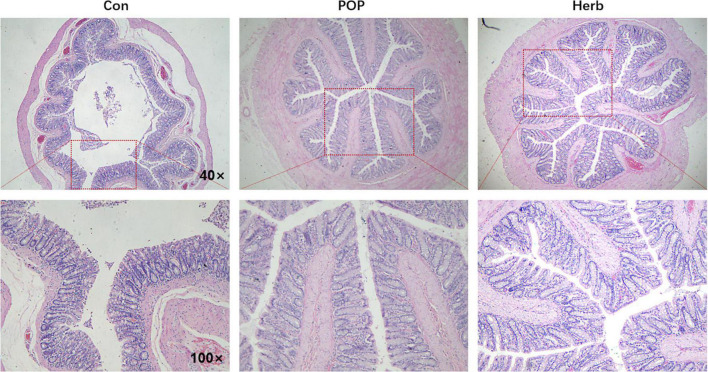
H&E staining of the colon. Regular morphology and clearer and more intact structures of the colons of rats were observed in the POP and herb groups than in the Con group.

Figure 3 shows regular morphology and clearer and more intact structures of the colons of rats in the POP and herb groups than in the Con group, and POP supplementation promoted intestinal villi development.

### Changes in the microbiota structure of fecal samples

**FIGURE 4:**
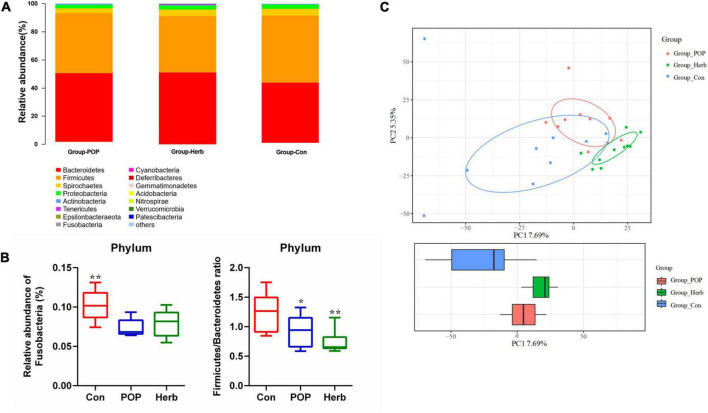
**(A,B)** Changes in the structure of fecal microbiota at the phylum level in the Con, POP, and herb groups. **(C)** PCA plots of all samples. * and ** indicate statistically significant differences (*P* < 0.05 and *P* < 0.01, respectively).

In all samples, the coverage rate of goods was >98.3%, indicating that the sequencing depth of the fecal microbiota analysis met the experimental requirements. In the alpha diversity analysis, the Chao1 and observed indices reflected the degree of bacterial abundance, whereas Shannon and Simpson indices were used to evaluate the microbial community’s diversity. As shown in Table 1, POP and *P. oleracea* herb supplementation slightly increased the abundance of intestinal microbiota in aging rats but did not have an effect on diversity. According to the species annotation results, the dominant bacteria in each group at the phylum level were Bacteroidetes and Firmicutes. Other phyla, including Spirochaetes, Proteobacteria, Actinobacteria, and Tenericutes, showed a low relative abundance (Figure 4A). Compared to the Con group, POP and purslane-supplementation significantly reduced the relative abundance of Fusobacteria and Firmicutes/Bacteroidetes (F/B) ratio (Figure 4B). Principal component analysis showed that the clustering effect of samples in the herb and POP groups was better, while there were large differences between samples in the Con group. There was a significant difference between the treatment and Con groups (Figure 4C).

**TABLE 1:** Alpha-diversity indices of the fecal microbiota.

Sample	Chao1	Goods-coverage	Observed	Shannon	Simpson
Con	3,972.63 ± 146.36^a^	0.9836 ± 0.0006^b^	2,973.89 ± 127.86^c^	7.87 ± 0.32^d^	0.976 ± 0.011^e^
POP	4,104.48 ± 228.89^a^	0.9831 ± 0.0010^b^	3,060.96 ± 207.71^c^	7.86 ± 0.25^d^	0.977 ± 0.009^e^
Herb	4,007.11 ± 150.93^a^	0.9835 ± 0.0007^b^	2,977.06 ± 139.28^c^	7.69 ± 0.18^d^	0.971 ± 0.005^e^

Values are represented as the means ± SD. Different letters indicate significant differences.

### Analysis of differences in species composition

**FIGURE 5:**
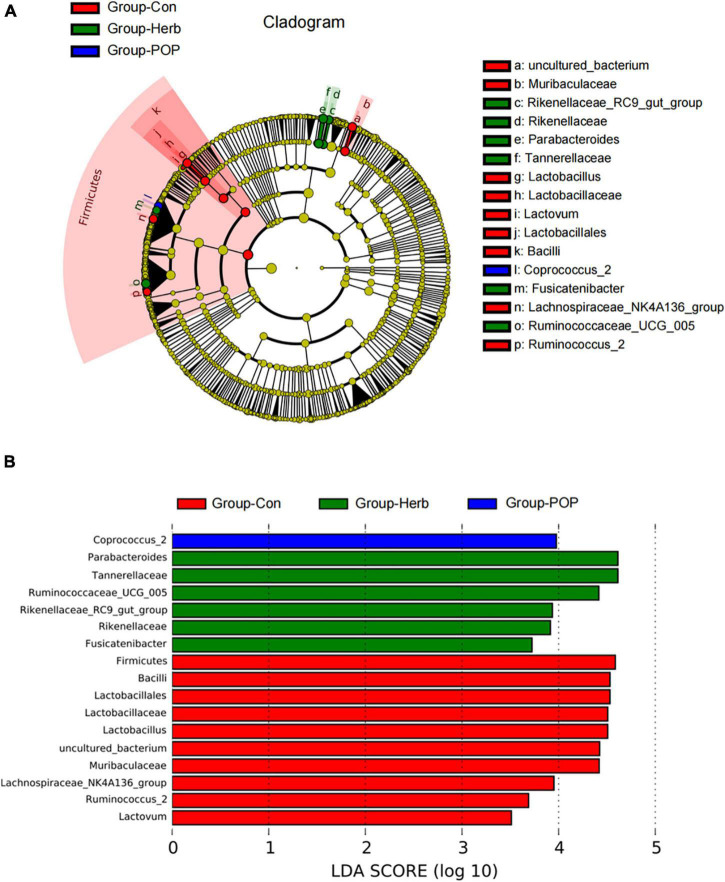
**(A)** LEfSe multilevel species hierarchy tree. **(B)** Microbial populations with an LDA score >3.0.

LEfSe analysis was used to identify differences in species composition in each group’s fecal microbiota. The results showed that the relative abundance of *Coprococcus*_2 was significantly enriched after POP supplementation, and the relative abundance of the *Rikenellaceae*_RC9_gut_group, *Rikenellaceae*, *Parabacteroides*, Tannerellaceae, *Fusicatenibacter*, and *Ruminococcaceae*-UCG-005 was significantly increased after purslane supplementation. However, in the Con group, the relative abundance of uncultured bacteria, *Muribaculaceae*, *Lactobacillus*, *Lactobacillaceae*, *Lactobacillales*, *Lactovum*, *Bacilli Lachnospiraceae*-NK4A136, and *Ruminococcus*-2 groups were significantly increased (Figure 5A,B).

### Metabolite profiling changes after *Portulaca oleracea* polysaccharide treatment

**FIGURE 6:**
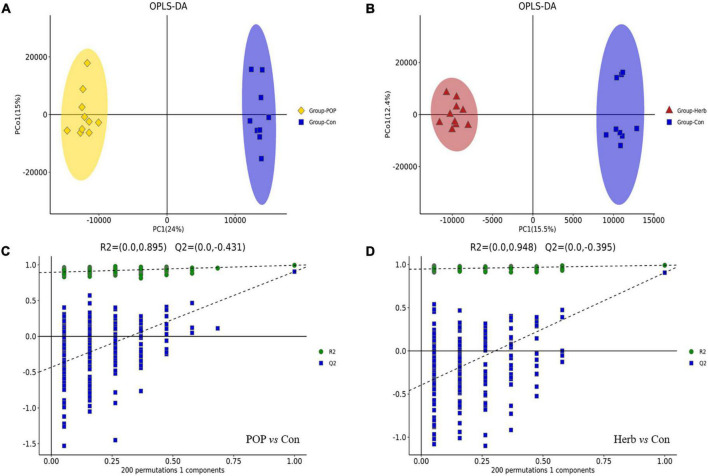
**(A,B)** OPLS-DA score lots of fecal metabolites in each group profiled through LC-MS. **(C)** Permutation tests for the OPLS-DA model in the POP and Con groups. **(D)** Permutation tests for the OPLS-DA model in the herb and Con groups.

A total of 14,412 metabolites were identified from the METLIN, Human Metabolome Database, lipidmaps (v. 2.3), and the self-built databases. The names, retention times, formulas, and *m/z* of the fecal metabolites in both positive and negative ion modes are shown in Supplementary Table 1. After data processing of all groups by QI, 6,293 and 8,119 ions were detected in the negative and positive modes, respectively. The score scatter plots for the OPLS-DA models showed clear differentiations among the POP, herb, and Con groups (Figures 6A,B). The OPLS-DA results indicated that the R2Y and Q2 values in the POP and Con groups were 0.994 and 0.905, respectively, while the R2Y and Q2 values in the herb and Con group were 0.992 and 0.906, respectively. This indicates that the existing model’s prediction was stable and accurate. Furthermore, sevenfold cross validation and response permutation testing were used to investigate the quality of the model (Figures 6C,D). Notably, the Q2 values in both analyses were negative (−0.431 and −0.395), indicating a low risk of overfitting.

Based on the VIP-plot analysis results, the ions with VIP > 1 and *P* < 0.05 were selected as differential metabolites, among which, there were 1,631 differences (*P* < 0.05) and 747 differences in lipids and lipoid-like molecule classifications in POP vs. Con analysis. There were 1,671 differential metabolites in Herb vs. Con (*P* < 0.05) analysis, and 684 differences in the lipid and lipoid-like molecule classifications. After adjusting the screening conditions to a score >50, VIP > 1.0, and *P* < 0.05, POP and purslane supplementation significantly regulated 33 lipids and function-like metabolites related to aging in rats, of which nine were up-regulated and 24 were down-regulated (Table 2).

**TABLE 2:** The 33 kinds of lipid and lipid molecular metabolites that significantly influenced by purslane polysaccharides in aging rats.

Common name	POP/ Con	Herb/ Con	*m/z*	Formula	Class	Adducts	Rt (min)	HMDB
5beta-Cholestane-3alpha,7alpha,12alpha,25-tetrol	↓**	↓**	459.3464	C_27_H_48_O_4_	Steroids and steroid derivatives	M + Na	11.24	HMDB0000524
5-Androstene-3b,16b,17a-triol	↓**	↓**	351.2176	C_19_H_30_O_3_	Steroids and steroid derivatives	M + FA-H	8.80	HMDB0000523
5b-Cholestane-3a,7a,12a,23-Tetrol	↓**	↓**	459.3465	C_27_H_48_O_4_	Steroids and steroid derivatives	M + Na	11.74	HMDB0001968
Soyasaponin I	↓**	↓**	943.5248	C_48_H_78_O_18_	Prenol lipids	M + H, M + Na	9.03	HMDB0034649
Soyasapogenol E	↑**	↑**	479.3483	C_30_H_48_O_3_	Prenol lipids	M + Na	8.02	HMDB0034652
beta-Elemolic acid	↑**	↑**	479.3480	C_30_H_48_O_3_	Prenol lipids	M + Na	7.82	HMDB0034961
3alpha-3-Hydroxytirucalla-7,24-dien-21-oic acid	↑**	↑**	479.3482	C_30_H_48_O_3_	Prenol lipids	M + Na	8.31	HMDB0034962
Medicagenic acid	↓**	↓**	501.3224	C_30_H_46_O_6_	Prenol lipids	M-H, M + FA-H	10.30	HMDB0034551
(3beta,17alpha,23S)-17,23-Epoxy-3,29-dihydroxy-27-norlanost-8-en-24-one	↓*	↓**	459.3468	C_29_H_46_O_4_	Prenol lipids	M + H	11.02	HMDB0035142
Oxysolavetivone	↓**	↓**	279.1601	C_15_H_22_O_2_	Prenol lipids	M + FA-H	7.29	HMDB0034956
(1alpha,4beta,5beta)-4-Hydroxy-7(11),10(14)-guaiadien-8-one	↓**	↓**	235.1690	C_15_H_22_O_2_	Prenol lipids	M + H-H_2_O, M + H	8.65	HMDB0036449
Tuberonone	↓**	↓*	219.1741	C_15_H_24_O_2_	Prenol lipids	M + H-H_2_O	10.53	HMDB0039688
3,14-Dihydroxy-11,13-dihydrocostunolide	↓**	↓**	267.1589	C_15_H_22_O_4_	Prenol lipids	M + H-H_2_O, M + H	7.93	HMDB0035647
20,24-Epoxy-25,26-dihydroxydammaran-3-one	↓*	↓**	457.3673	C_30_H_50_O_4_	Prenol lipids	M + H-H_2_O, M + H	11.08	HMDB0039692
alpha,gamma-Onoceradienedione	↓*	↓**	439.3567	C_30_H_46_O_2_	Prenol lipids	M + H-H_2_O, M + H	12.66	HMDB0036787
22alpha-Hydroxyerythrodiol	↑**	↑**	481.3635	C_30_H_50_O_3_	Prenol lipids	M + Na	7.96	HMDB0034549
Camelledionol	↓**	↓**	423.3243	C_29_H_44_O_3_	Prenol lipids	M + H-H_2_O	10.58	HMDB0035730
Soyasaponin II	↓*	↓*	913.5143	C_47_H_76_O_17_	Prenol lipids	M + H, M + Na	9.33	HMDB0034650
(4S,8R)-8,9-Dihydroxy-p-menth-1(6)-en-2-one	↑**	↑**	229.1076	C_10_H_16_O_3_	Prenol lipids	M + FA-H	5.70	HMDB0039052
Absindiol	↓**	↓*	267.1588	C_15_H_22_O_4_	Prenol lipids	M + H-H_2_O, M + H	9.47	HMDB0033229
Camelliagenin A	↑**	↑**	497.3584	C_30_H_50_O_4_	Prenol lipids	M + Na	7.32	HMDB0034528
alpha-Rotunol	↓**	↓**	279.1600	C_15_H_22_O_2_	Prenol lipids	M + FA-H	8.69	HMDB0035869
4a-Methylzymosterol-4-carboxylic acid	↓*	↓**	443.3517	C_29_H_46_O_3_	Prenol lipids	M + H-H_2_O, M + H	11.43	HMDB0006927
(1beta,2beta,5beta)-p-Menth-3-ene-1,2,5-triol	↑**	↑**	231.1233	C_10_H_18_O_3_	Prenol lipids	M + FA-H	6.07	HMDB0038978
1-(8Z,11Z,14Z-Eicosatrienoyl)-glycero-3-phosphate	↓*	↓**	478.2926	C_23_H_41_O_7_P	Glycerophospholipids	M + NH_4_	11.30	HMDB0062313
MG (24:0/0:0/0:0)	↑**	↑**	481.3634	C_27_H_54_O_4_	Glycerolipids	M + K	8.08	HMDB0011588
MG (0:0/18:2(9Z,12Z)/0:0)	↓*	↓**	355.2838	C_21_H_38_O_4_	Fatty acyls	M + H	12.74	HMDB0011538
20-HDoHE	↓**	↓*	327.2313	C_22_H_32_O_3_	Fatty acyls	M + H-H_2_O	7.46	HMDB0060048
Vaccenic acid	↓*	↓*	300.2893	C_18_H_34_O_2_	Fatty acyls	M + H-H_2_O, M + NH_4_	12.57	HMDB0003231
Undecylenic acid	↓**	↓*	185.1535	C_11_H_20_O_2_	Fatty acyls	M + H-H_2_O, M + H	8.08	HMDB0033724
Muricin A	↑**	↑*	641.4635	C_35_H_64_O_7_	Fatty acyls	M + FA-H	10.80	HMDB0036977
(Z)-6-Tetradecene-1,3-diyne-5,8-diol	↓**	↓**	265.1444	C_14_H_20_O_2_	Fatty acyls	M + FA-H	7.93	HMDB0038996
Undecanedioic acid	↑**	↑**	261.1341	C_11_H_20_O_4_	Fatty acyls	M + FA-H	5.39	HMDB0000888

Score >50, VIP >1.0, **P < 0.01, *P < 0.05.

The symbols ↑ and ↓ mean the metabolite is up and downregulated in POP and herb groups compared with the Con group. Significant difference compared with the Con group.

### Changes in fecal metabolites and corresponding pathways

**FIGURE 7:**
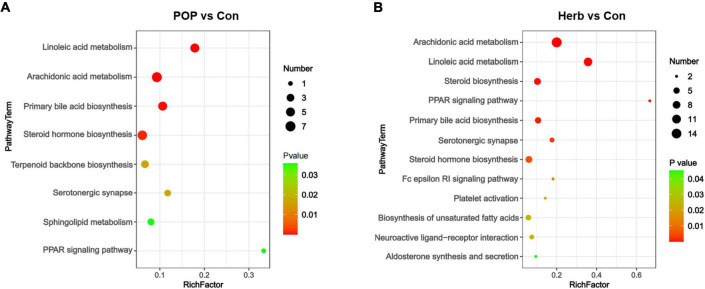
Changes in the corresponding pathways associated with fecal metabolites. **(A)** Major changes between the POP and Con groups (*P* < 0.05). **(B)** Major changes between the herb and Con groups (*P* < 0.05).

To understand metabolic pathway changes in different samples, we conducted metabolic pathway enrichment analyses for differential metabolites based on the KEGG database. The results showed that the main changes between the POP and Con groups included linoleic acid (LA) metabolism, arachidonic acid metabolism, primary bile acid biosynthesis, steroid hormone biosynthesis, terpenoid backbone biosynthesis, serotonergic synapse, sphingolipid metabolism, and the peroxisome proliferator-activated receptor (PPAR) signaling pathways (Figure 7A). Changes between the herb and Con groups included arachidonic acid metabolism, LA metabolism, steroid biosynthesis, the PPAR signaling pathway, primary bile acid biosynthesis, serotonergic synapse, steroid hormone biosynthesis, the Fc epsilon RI signaling pathway, platelet activation, biosynthesis of unsaturated fatty acids, neuroactive ligand-receptor interaction, and aldosterone synthesis and secretion (Figure 7B).

### Correlation among serum lipids, fecal metabolites, and intestinal microbiota

**FIGURE 8:**
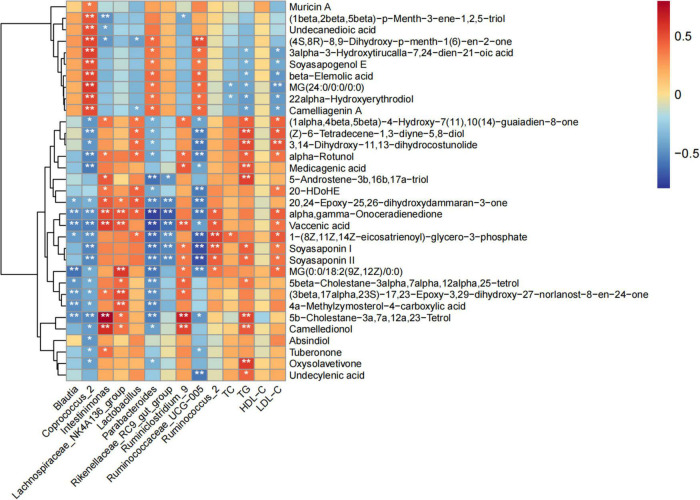
Correlation between serum lipids, fecal metabolites, and gut microbiota in Spearman’s correlation coefficient. * and ** indicate statistically significant differences (*P* < 0.05 and *P*<0.01, respectively).

The Spearman correlation coefficient was used to analyze the correlation between serum lipid level, fecal metabolites improved by POPs, and intestinal microbiota (based on the LEfSe analysis). The results showed that the main fecal metabolites improved by POPs were significantly correlated with intestinal microbiota. The *Coprococcus*-2 enriched by POPs was negatively correlated with oxysolavetivone, absindiol, camelledionol, 5b-cholestane-3a,7a,12a,23-tetrol, 4a-methylzymosterol-4-carboxylic acid, (3beta,17alpha,23S)-17,23-epoxy-3,29-dihydroxy-27-norlanost-8-en-24-one, 5beta- cholestane-3alpha,7alpha,12alpha,25-tetrol, MG[0:0/18:2(9Z, 12Z)/0:0], soyasaponin II, soyasaponin I,1-(8Z,11Z,14Z-eicosatrienoyl)-glycero-3-phosphate, vaccenic acid, alpha, gamma-onoceradienedione, 20,24-epoxy-25,26-dihydroxyda mmaran-3-one, medicagenic acid, alpha-rotunol, 3,14-dihyd roxy-11,13-dihydrocostunolide, (Z)-6-tetradecene-1,3-diyne-5,8-diol, and (1alpha,4beta,5beta)-4-hydroxy-7(11),10(14)-guaiadien-8-one. The *Coprococcus*_2 enriched by POPs was positively correlated with camelliagenin A, 22alpha-hydroxyerythrodiol, MG(24:0/0:0/0:0), beta-elemolic acid, soyasapogenol E, 3alpha-3-hydroxytirucalla-7,24-dien-21-oic acid, (4S,8R)-8,9-dihydroxy-p-menth-1(6)-en-2-one, undecanedioic acid, (1beta,2beta,5beta)-p-menth-3-ene-1,2,5-triol, and muricin A. Furthermore, TC was positively correlated with 1-(8Z,11Z,14Z-eicosatrienoyl)-glycero-3-phosphate, and negatively correlated with 22alpha-Hydroxyerythrodiol and MG (24:0/0:0/0:0). TG and LDL-C were inversely associated with 3alpha-3-hydroxytirucalla-7,24-dien-21-oic acid, soyasapogenol E, beta-elemolic acid, MG (24:0/0:0/0:0),22alpha-hydroxyerythrodiol and camelliagenin A, and positively correlated with alpha-rotunol, 3,14-dihydroxy-11,13-dihydrocostunolide, (Z)-6-tetradecene-1,3- diyne-5,8-diol,(1alpha,4beta,5beta)-4-hydroxy-7(11),10(14)-guaiadien-8-one, and soyasaponin I and II. TG was positively associated with undecylenic acid, oxysolavetivone, camelledionol, 5b-cholestane-3a,7a,12a,23-Tetrol, 5beta- cholestane-3alpha,7alpha,12alpha,25-tetrol, 5-androstene-3b, 16b,17a-triol, and medicagenic acid. LDL-C was positively associated with MG [0:0/18:2(9Z,12Z)/0:0], 1-(8Z,11Z,14Z-eicosatrienoyl)-glycero-3-phosphate, vaccenic acid, alpha, gamma-onoceradienedione, and 20-HDoHE (Figure 8). These correlation results indicate that serum lipids, fecal metabolites, and intestinal microbiota in the POP-treated environment constitute a triangular relationship of mutual influence. Furthermore, changes in serum lipid levels induced by POPs might alter host’s metabolism.

## Discussion

Aging is associated with multiple systemic dysfunctions, lipid metabolism disorders, and chronic inflammatory states, leading to ASCVD (1). Dyslipidemia is one of the most important triggers of atherosclerosis in the arterial wall and ASCVD, which is characterized by abnormal elevated levels and deposition of apolipoprotein B, including LDL-C (20). Previous studies have shown that POP has biological properties, such as anti-tumor, anti-oxidation, anti-diabetes, anti-viral, and intestinal microbiota regulation (21–24). Therefore, to investigate the mechanism by which purslane polysaccharides improve serum lipid levels in aging rats, we studied the intestinal metabolite profile and changes in the microbial community structure.

As expected, POPs improved dietary volume and weight loss in the aging rats. High levels of plasma glucose, TG, TC, and LDL-C reflect the state of lipid absorption and metabolism, which contribute the risk of various diseases (25). However, POPs can regulate plasma glucose, TG, TC, and LDL-C levels and reduce these potential risks. Gong et al. also found that POPs significantly reduced fasting blood glucose, TC, and TG levels, and increased HDL-C levels, body weight, and serum insulin levels in diabetic mice (26). The liver regulates lipid homeostasis and participates in glucose synthesis and metabolism (27). As important indicators of liver function, ALT and γ-GT are related to the lipid profile of patients with non-alcoholic fatty liver disease (28). Interestingly, we found that POPs significantly reduced ALT and γ-GT levels aging rats. Zheng et al. confirmed that purslane markedly decreases liver injury effects by reducing serum glucose, anti-aspartate aminotransferase, ALT, TG, and TC levels in diabetic mice (29). Our results suggest that purslane may reduce fasting blood glucose, TG, and ALT levels and alleviate the effects of weight loss in aging rats. These results suggest that POPs are one of the main active components of purslane that lowers blood lipid levels and protects the liver. Additionally, our study showed that POPs could alleviate the effects of cardiac hypertrophy in elderly rats. However, whether purslane polysaccharides can improve blood lipid levels and protect the heart requires further investigation. Therefore, POPs may be a promising factor in the prevention of dyslipidemia.

Food and intestinal microbiota interact to processes dietary nutrients into metabolites. Diet also affects intestinal microbiota composition (30). Due to the close anatomical and functional interaction of the gut-liver axis, the microbiota-gut-liver-brain axis has drawn great attention in recent years, and a growing number of studies have shown that gut microbiota is linked with many diseases, such as depression, autism, anxiety, obesity, schizophrenia, diabetes, Parkinson’s disease (PD) and Alzheimer’s disease (31). The gut microbiota composition in the elderly individuals is characterized by decreases in diversity and the abundance of butyrate-producing species (32). Compared with the Con group, the Chao1 and observed indices of the POP group increased by 3.32 and 2.93% in the α-diversity analysis, respectively, suggesting that POPs potentially improve the abundance of intestinal microbiota in aging rats. Moreover, β-diversity showed significant differences within the Con group, while POP and herb group cohesion was good, suggesting that *P. oleracea* or its polysaccharide-active substances, affects the composition of intestinal microbiota in elderly rats. At the phylum level, a higher F/B ratio is positively correlated with energy acquisition, which may be a risk factor for metabolic syndrome (33). In this study, a significant change in the gut microbiota was observed, and the F/B ratio in the Con group was significantly higher than that in the POP and herb groups, suggesting that POPs and purslane may reduce energy acquisition in aging rats and help prevent metabolic syndrome. Fusobacteria, at the phylum level, is an intestinal microbiota associated with colorectal cancer (34, 35). Here, POP and purslane supplementation significantly reduced the relative abundance of Fusobacteria in aging rats. Studies in diabetic populations have shown that *Fusobacteria* are negatively correlated with higher dietary fiber intake (36). Notably, this risk was also reduced by POP and purslane feeding.

It is widely believed that Lactobacillaceae, at the family level, and *Lactobacillus*, at the genus level, are beneficial in improving human health (37). Recent studies have reported higher levels of *Lactobacillaceae* in obese mice and patients (38). Moreover, a high abundance of lactic acid bacteria was positively correlated with obesity-related characteristics (39). A recent study suggests that some species of lactic acid bacteria, such as *Lactobacillus reuteri*, are associated with obesity (40). In the present study, the relative abundance of *Lactobacillaceae* and *Lactobacillus* were significantly higher in the Con group than in the other two groups. Furthermore, the levels of *Lactovum*, *Bacilli*, *Lachnospiraceae*-NK4A136, and *Ruminococcus*-2 in Firmicutes, and *Muribaculaceae* in Bacteroidetes phylum in the Con group were significantly higher than those in the POP and herb groups. High-fat/carbohydrate diets program the gut microbiota to be dominated by *Firmicutes* (*Clostridium*), *Prevotella*, and *Methanobrevibacter* (41). *Muribaculaceae* were the dominant genus in the intestinal microbiota of mice fed a high-fat diet (42). As expected, *P. oleracea* and POPs inhibited the proliferation of these bacteria, which may help prevent dyslipidemia. *Coprococcus* spp. is fungus capable of fermenting complex plant carbohydrates in the Lachnospiraceae family (43). However, *Coprococcus* abundance is low in patients with PD) and children with autism spectrum disorders (44, 45). In this study, the relative abundance of *Coprococcus-*2 was significantly higher in the intestines of aging rats. Prior studies have found that POPs significantly improve the γ-aminobutyric acid level in juvenile rat serum (data not shown). These results suggest that POPs may be beneficial because they metabolize complex plant carbohydrates in the gut. Mushroom-enriched diets modulate the microbial-gut-brain axis in mice and reduce oxidative stress in the brain (46). Increased *Tannerellaceae* may positively influence the composition of the intestinal microbiota. A study of normal-weight subjects who consumed 40 g/day of resistant starch suggested that the relative abundance of *Ruminococcaceae*-UCG-005 was significantly increased (47). High amounts of *Rikenellaceae* are protective against cardiovascular and metabolic diseases related to visceral fat and, is thus a potential marker of healthy aging, and possibly, longevity (48). However, the results of bacterial community changes from PD patients’ fecal samples showed that the abundance of the *Rikenellaceae*-RC9-gut group and *Parabacteroides* was relatively high (49). Frailty is a critical aging-related syndrome, but the decreased relative abundance of *Fusicatenibacter* was found in frailty (50). In this study, supplementation with *P. oleracea* increased the relative abundance of *Parabacteroides*, *Tannerellaceae*, and *Fusicatenibacter*, and *Rikenellaceae*-RC9-gut-group abundance in *Ruminococcaceae*-UCG-005 and *Rikenellaceae*, suggesting that POPs were more specific in fostering intestinal microbiota in aging rats than those fostered by *P. oleracea*, which may be related to the complex composition of *P. oleracea*.

We used the LC-MS/MS metabonomic method to analyze metabolite changes in the feces of aging rats fed POPs and *P. oleracea*. In the rats, lipids and lipoid-like molecules, including steroids and steroid derivatives, prenol lipids, glycerophospholipids, glycerolipids, and fatty acids, were significantly altered. Supplementation with POPs and purslane improved lipid metabolism in aging rats by regulating related pathways. Our results showed that POPs and purslane regulate related metabolic pathways in aging rats, with the PPAR signaling pathway being the most affected, followed by LA metabolism. Purslane is an excellent source of ω-3 fatty acids, including α-linolenic acid and LA, which are essential for normal growth, health promotion, and disease prevention in humans (51). The metabolites levels derived from LA, such as 10-hydroxy-cis-12-octadecenoic acid, were significantly reduced in the intestinal tract of rats fed a high-fat diet for 2 weeks, but were reversed by supplementation with LA (52). During adipocytes differentiation, some transcription factors, including CCAAT/enhancer binding proteins and PPAR-γ, activate fat production. Studies have found that citrus flavonoids inhibit intracellular TG and fat accumulation, and reduce PPAR-γ2 expression (53). The leukotriene family is a metabolite catalyzed by arachidonic acid through 5-lipoxygenase, and is an important indicator of the inflammatory response in atherosclerosis (54). In this study, 12-keto-tetrahydro-leukotriene B4 was enriched in the PPAR signaling pathway in the POP and herb groups. POPs and purslane supplementation significantly reduced 12-keto-tetrahydro-leukotriene B4 levels (Supplementary Tables 2, 3). Therefore, POPs in *P. oleracea* may improve serum lipid levels in aging individuals mainly by regulating the PPAR signaling pathways and LA metabolism.

Moreover, in our study, 5beta-cholestane-3alpha,7alpha,12alpha,25-tetrol, 5-androstene-3b,16b,17a-triol, and 5b-cholestane-3a,7a,12a,23-tetrol were classified as steroids and steroid derivatives. Cholesterol is a precursor to steroid production, and dysfunction in cholesterol transport and/or steroid metabolism can lead to lipid accumulation with deleterious effects (55). Additionally, 5beta-cholestane-3alpha,7alpha,12alpha,25-tetrol, 5-androstene-3b,16b,17a-triol, and 5b-cholestane-3a,7a,12a,23-tetrol showed significant positive correlations with TG. POP and *P. oleracea* intake notably decreased 5beta-cholestane-3alpha,7alpha,12alpha,25-tetrol, 5-androstene-3b,16b,17a-triol, and 5b-cholestane-3a,7a,12a,23-tetrol levels. Moreover, 5beta-cholestane-3alpha,7alpha,12alpha,25-tetrol, and 5b-cholestane-3a,7a,12a,23-tetrol negatively correlated with *Coprococcus-2*, but exhibited a positive correlation with *Lachnospiraceae*-NK4A136 group. Li et al. found that, in an animal model of high-fat diet, *Coprococcus* was negatively correlated with cholesterol metabolic parameters after a supplemental diet of wood pulp-derived sterols (56). The results revealed that a decrease in steroids and steroid derivatives might benefit the lipid metabolism effect of POP by accelerating *Coprococcus* and inhibiting *Lachnospiraceae-*NK4A136 expression. Vaccenic acid is regulated by fatty acid metabolism. Interestingly, vaccenic acid levels were higher in men with hyperglycemic-hyperinsulinemic than in men with normoglycemic-normoinsulinemic, and were positively associated with both fasting insulin and homeostatic model assessment for insulin resistance (57). It was significantly higher in the Con group than in the POP and herb groups, indicating that POPs and purslane can effectively regulate long-chain fatty acids to improve lipid metabolism in aging rats. Furthermore, 1-(8Z,11Z,14Z-eicosatrienoyl)-glycero-3-phosphate is classified as a glycerophospholipid and positively correlated with *Lactobacillus*, TC, and LDL-C. After supplementation with L. acidophilus ZLA012, metabolites associated with lipid metabolism exhibited the highest enrichment relative to the Con group, and were mainly involved in glycerophospholipid, arachidonic acid, and LA metabolism (58). Biologically related prenol lipids include fat-soluble vitamins (i.e., vitamins A, E, and K) and antioxidant molecules such as carotenoids, and ubiquinones (59). In our study, soyasapogenol E, beta-elemolic acid, 3alpha-3-hydroxytirucalla-7,24-dien-21-oic acid, and 22alpha-hydroxyerythrodiol were involved in prenol lipid metabolism, which was significantly up-regulated by POPs and *P. oleracea*, while soyasaponins I and II were down-regulated. Studies have shown that soyasapogenol B exerts anti-obesity and anti-diabetic effects on adipocytes via lowering cellular TG levels by accelerating TG lipolysis and reducing resistin secretion (60). Our results showed that soyasapogenol E was significantly negatively correlated with TG and LDL-C, but positively correlated with *Ruminococcaceae*-UCG-005, *Parabacteroides*, and *Coprococcus*-2. Soyasaponins reportedly exert several functions, such as antioxidative, cholesterol-lowering, and anti-obesity activities. However, the bioavailability of soyasapogenol (aglycone type) was better than that of soyasaponin (glycoside type) (61). Furthermore, triterpenoids such as beta-elemolic acid, 22alpha-hydroxyerythrodiol and camelliagenin A, elemi resin component 3alpha-3-hydroxytirucalla-7, and 24-dien-21-oic acid, showed significant negative correlations with TG and LDL-C, while positively correlating with *Ruminococcaceae*-UCG-005, *Parabacteroides*, and *Coprococcus*-2. Thus, we speculated that POPs and purslane may facilitate the conversion of soyasaponins I and II into soyasapogenol E, increasing beta-elemolic acid, 22alpha-hydroxyerythrodiol, camelliagenin A, 3alpha-3-hydroxytirucalla-7, and 24-dien-21-OIC acid levels and promoting *Ruminococcaceae*-UCG-005, *Parabacteroides*, and *Coprococcus*-2 expression, which were beneficial to TG lipolysis in aging rats. Glycerolipids form the body’s largest energy stores and are a major lipid class comprised mainly of monoacylglycerols, diacylglycerols, and triacylglycerols. MG (24:0/0:0/0:0) is a monoacylglyceride that is a minor component of most plant and animal tissues, and 2-monoacylglycerols are the major end-products of the intestinal digestion of dietary fats in animals via the pancreatic lipase enzyme (62). In this study, MG (24:0/0:0/0:0) was classified as a glycerolipid, and its levels significantly increased by POP and *P. oleracea* treatment. Although MG (24:0/0:0/0:0) was negatively correlated with TC, TG, and LDL-C, it was positively correlated with *Ruminococcaceae*-UCG-005, *Parabacteroides*, and *Coprococcus*-2. These results suggest that MG (24:0/0:0/0:0) may be a potentially beneficial metabolite of POPs and purslane to stabilize lipid levels in the elderly population and has a close relationship with *Ruminococcaceae*-UCG-005, *Parabacteroides*, and *Coprococcus*-2. Thus, POPs, and *P. oleracea* have similar but different beneficial effects on lipid levels in aging rats, and the effect of POPs was better than that of purslane, which might be related to the complex components of *P. oleracea*.

## Conclusion

Our study investigated the effect of POPs on lipid levels in aging rats, based on its effect on intestinal microbiota and their related metabolites. The results showed that POPs can promote the growth of *Coprococcus*-2, a complex plant carbohydrate-degrading bacterium, and reduce the abundance of some obesity-related bacteria, including *Lactobacillus* and *Muribaculaceae*. The improvement of the gut microbiomes affects the production of bacteria-derived metabolites, such as the decrease in 5beta-cholestane-3alpha,7alpha,12alpha,25-tetrol, and vaccenic acid levels in fatty acyls in steroids and steroid derivatives and increase of soyasapogenol E level in prenol lipids, and MG (24:0/0:0/0:0) in glycerolipids. Therefore, these results suggest that the effect of POPs on lipid levels in aging rats is related to the regulation of the intestinal microbiota and its metabolites.

## Data availability statement

The datasets presented in this study can be found in online repositories. The names of the repository/repositories and accession number(s) can be found below: https://www.ncbi.nlm.nih.gov/, PRJNA858605.

## Ethics statement

The animal study was reviewed and approved by the Institutional Animal Use and Care Committee of Jinggangshan University.

## Author contributions

QF and XH conceived and designed the experiment and drafted the manuscript. HH, AD, and ZY performed the experiments and collected the data. GF, XH, and YSH provided the resources and reviewed the manuscript. YPH and XH assisted with the interpretation of the data and checked the statistical analyses. All authors have made substantial contributions to the conception and design of the project and critically revised and approved the final submitted version of the manuscript.
